# SCF^β-TRCP^ regulates osteoclastogenesis via promoting CYLD ubiquitination

**DOI:** 10.18632/oncotarget.1971

**Published:** 2014-05-14

**Authors:** Xiaomian Wu, Hidefumi Fukushima, Brian J. North, Yoshiyuki Nagaoka, Katsuyuki Nagashima, Feng Deng, Koji Okabe, Hiroyuki Inuzuka, Wenyi Wei

**Affiliations:** ^1^ Chongqing key Laboratory for Oral Diseases and Biomedical Sciences, The Affiliated Hospital of Stomatology, Chongqing Medical University, Chongqing, P.R. China; ^2^ Department of Pathology, Beth Israel Deaconess Medical Center, Harvard Medical School, Boston, MA; ^3^ Department of Physiological Sciences and Molecular Biology, Fukuoka Dental College, Fukuoka, Japan

**Keywords:** β-TRCP, CYLD, tumor suppressor, degradation, phosphorylation, ubiquitination

## Abstract

CYLD negatively regulates the NF-κB signaling pathway and osteoclast differentiation largely through antagonizing TNF receptor-associated factor (TRAF)-mediated K63-linkage polyubiquitination in osteoclast precursor cells. CYLD activity is controlled by IκB kinase (IKK), but the molecular mechanism(s) governing CYLD protein stability remains largely undefined. Here, we report that SCF^β-TRCP^ regulates the ubiquitination and degradation of CYLD, a process dependent on prior phosphorylation of CYLD at Ser432/Ser436 by IKK. Furthermore, depletion of β-TRCP induced CYLD accumulation and TRAF6 deubiquitination in osteoclast precursor cells, leading to suppression of RANKL-induced osteoclast differentiation. Therefore, these data pinpoint the IKK/β-TRCP/CYLD signaling pathway as an important modulator of osteoclastogenesis.

## INTRODUCTION

Bone is consistently renewed throughout life by the opposing activities of osteoblastic bone formation and osteoclastic bone resorption pathways. Although osteoclasts are required for bone remodeling, excess activity of osteoclasts can lead to various diseases such as periodontal disease, osteoporosis, rheumatoid arthritis, multiple myeloma and metastatic cancers [1-7]. Osteoclasts are largely generated from differentiating bone marrow precursor cells, a process that is tightly regulated by various osteoclast specific factors. Importantly, as expressions of these key osteoclast regulators are controlled by the NF-κB signaling pathway, elucidating how NF-κB signaling is fine tuned during osteoclast differentiation has drawn increased research attention. To this end, the identification of osteoprotegerin and receptor activator nuclear factor-κB ligand (RANKL) as the dominant mediators of osteoclastogenesis has been a major advancement in our understanding of the osteoclast differentiation process [8]. Notably, RANKL specifically and potently activates nuclear factor of activated T cells cytoplasmic 1 (NFATc1), a key transcription factor for osteoclastogenesis and the master regulator of osteoclast differentiation, via targeting both the TRAF6/NF-κB and c-fos signaling pathways [9-13].

SCF (Skp1-Cullin1-F-box protein) E3 ligases are well studied Cullin-based E3 ligases, containing a holo-enzyme composed of three static subunits, namely Skp1 (S-phase kinase-associated protein-1), Cullin 1 and Rbx1/Roc 1, as well as a variable F-box protein subunit [14]. To date, 69 putative F-box proteins have been identified in the human genome, but the substrates and functions of most F-box proteins have not yet to be fully characterized [14]. The beta-transducin repeat-containing protein (β-TRCP) is one of the most well characterized F-box proteins, which confers substrate specificity to the SCF^β-TRCP^ E3 ligase complex. β-TRCP regulates diverse cellular processes largely by promoting degradation of its substrates in a phosphorylation-dependent manner [15]. Notably, SCF^β-TRCP^ activity has been characterized as a critical positive regulator of NF-κB signaling, both by promoting the processing and activation of NF-κB1 and NF-κB2, as well as promoting the degradation of IκB, an endogenous inhibitor of NF-κB [16, 17].

Ubiquitination is a reversible post-translational modification where E3 ubiquitin ligases conjugate ubiquitin moieties, either as mono-ubiquitination or different linkage-specific poly-ubiquitinated chains, to the targeted substrates. The activities of various ubiquitin ligases can be opposed by deubiquitinating enzymes (DUBs). Cylindromatosis (also designated as CYLD) is a well-characterized DUB [18], where mutations in CYLD are reported to cause cylindramotosis implicating CYLD as a tumor suppressor [19]. Besides suppressing tumorigenesis, CYLD has also been found to play critical roles in the immune response and bone remodeling largely via regulating the NF-κB signaling pathway [19-25]. Notably, multi-layer regulation of the timely activation of the NF-κB pathway has been shown to be governed by ubiquitin-dependent processes. In this regard, while β-TRCP is reported as a positive regulator, CYLD is considered largely as a negative regulator of the NF-κB pathway by antagonizing TRAF-mediated K63-linkage polyubiquitination of various substrates [16, 26-28]. Recent studies have demonstrated that under diverse stimulations, TRAFs-mediated K63-linkage ubiquitination, as well as LUBAC-dependent linear-ubiquitination of NEMO (IKKγ) [29], synergistically leads to timely activation of NF-κB kinase alpha (IKKα) and IKKβ to phosphorylate the IκBs. Subsequently, phosphorylated IκBs are degraded by SCF^β-TRCP^ to induce full NF-κB activation. Activated NF-κB in turn promotes transcriptional upregulation of CYLD, leading to the deubiquitination and thereby deactivation of TRAFs, functioning as a negative feedback loop between the NF-κB and CYLD signaling axis [27, 28].

Recent studies have suggested that CYLD negatively regulates osteoclastogenensis through deubiquitination of TRAF6 [30]. Consistently, CYLD deficient mice displayed an osteoporosis phenotype harboring a low bone density. Moreover, osteoclast precursors derived from CYLD deficient mice are more sensitive to RANKL-induced differentiation [30]. Notably, a mutant (P392L) of p62/SQSTM1, a multifunctional ubiquitin-binding protein that is necessary for receptor internalization and protein turnover, stimulates differentiation of osteoclast in Paget's disease, a disorder characterized by abnormal osteoclastogenensis [31]. Furthermore, mice carrying the p62/SQSTM1-P392L mutation show loss of binding between CYLD and TRAF6, and loss of deubiquitination activity of CYLD towards TRAF6, supporting the critical role of CYLD in osteoclast differentiation.

Given the critical role of CYLD in bone remodeling, it is important to further understand how CYLD abundance is controlled during physiological processes such as osteoclast differentiation. However, the identity of the upstream E3 ubiquitin ligase responsible for ubiquitinating CYLD has remained elusive. Here, we report that β-TRCP and IKK are critical mediators controlling CYLD protein degradation, and β-TRCP contributes to regulating osteoclastogenesis through negatively controlling CYLD protein stability. Our results further suggest that deregulation of the CYLD degradation pathway might lead to aberrant bone formation, resulting in bone related diseases.

## RESULTS

### CYLD interacts with the SCF^β-TRCP^ E3 ligase complex

**Figure 1:**
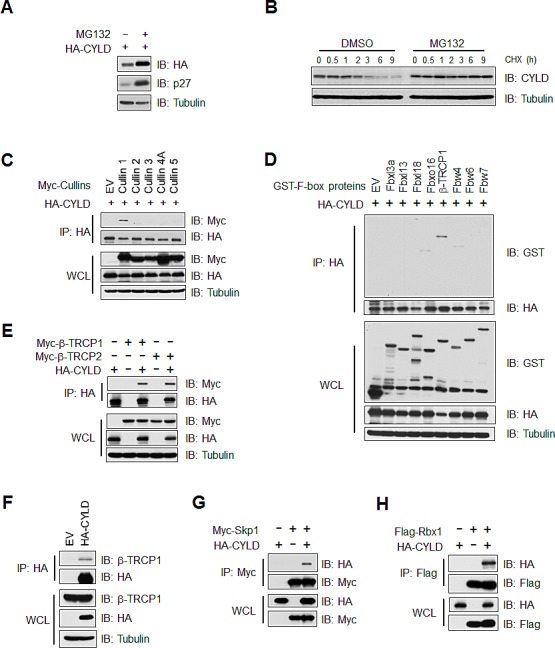
CYLD interacts with the SCF^β-TRCP^ E3 ligase complex (A) Immunoblot (IB) analysis of whole cell lysates (WCL) derived from DLD1 cells transfected with HA-CYLD with or without MG132 (15 μM) treatment. (B) RAW264.7 mouse macrophage cells were treated with RANKL (50 ng/ml) for 24 hours and then treated with or without MG132 (15 μM) for 6 hours prior to cycloheximide (CHX) treatment (20 μg/ml). At the indicated time points, WCL were prepared, and IB analysis was performed with the indicated antibodies. (C) IB analysis of WCL and anti-HA immunoprecipitates (IP) derived from 293T cells, in which HA-CYLD was co-transfected with empty vector, Myc-Cullin1, 2, 3, 4A or 5 expression plasmids. (D) IB analysis of WCLs and HA-IP derived from 293 cells transfected with HA-CYLD and the indicated GST-tagged F-box proteins. (E) IB analysis of WCL and anti-HA IP derived from 293T cells, in which HA-CYLD was co-transfected with Empty vector (EV), Myc-β-TRCP1 or Myc-TRCP2 plasmids as indicated. (F) IB of WCLs and anti-HA IP derived from 293T cells transfected with HA-CYLD or EV as a negative control. (G) IB analysis of WCL and anti-Myc IP derived from 293T cells transfected with HA-CYLD and Flag-Skp1 as indicated.(H) IB analysis of WCL and anti-Flag IP derived from 293T cells transfected with HA-CYLD and Flag-Rbx1 as indicated.

The stability of multiple critical components of the NF-κB pathway is tightly controlled in a proteasome-dependent manner. Hence, we first examined whether CYLD protein stability is also regulated by the ubiquitin-proteasome pathway. Since proteasome inhibitors, including MG132, have been reported to inhibit mRNA expression of CYLD and several NF-κB target genes in part by elevating the expression of IκB, an endogenous inhibitor of NF-κB (Supplementary Figures 1A-G) [27], we analyzed how the proteasome inhibitor MG132 might affect the abundance of exogenous CYLD. Under ectopic expression conditions, the transcription of exogenous CYLD mRNA is under control of the CMV promoter. Contrary to endogenous CYLD protein levels, we found that exogenous CYLD protein was upregulated in MG132 treated DLD1 cells (Figure 1A). Furthermore, MG132 treatment extended the half-life of endogenous CYLD protein following RANKL stimulation in RAW264.7 mouse macrophage cells, as RANKL stimulation induces CYLD mRNA expression, thereby bypassing the down-regulation of CYLD mRNA expression by MG132 (Figure 1B). These data suggests that CYLD protein abundance is subjected to the regulation by the proteasome degradation pathway.

As Cullin-Ring type complex forms the largest group of E3 ubiquitin ligases [32], we next tested which specific Cullin-Ring E3 ligase might be involved in the regulation of CYLD protein stability. Using a panel of Cullin members, we found that exogenous CYLD specifically interacted with Cullin-1, but not other Cullin family members (Figure 1C). As Cullin-1 is a scaffolding component of the SCF (Skp1-Cullin1-F-box protein) E3 ligase complex whereby the F-box protein confers substrate specificity, we continued to explore which F-box protein might interact with CYLD. To this end, using a panel of F-box proteins, we identified that CYLD specifically interacted with β-TRCP1, but not any of the other F-box proteins we tested (Figure 1D). Consistent with these observations, using co-immunoprecipitation experiments, we detected exogenously expressed CYLD associated with ectopically expressed β-TRCP1 and β-TRCP2 (Figure 1E) and endogenous β-TRCP1 (Figure 1F). Moreover, under extopic co-expression conditions, CYLD also interacted with the SCF E3 ligase components Skp1 (Figure 1G) and Rbx1 (Figure 1H), suggesting that the SCF^β-TRCP^ E3 ligase complex is involved in regulating CYLD protein stability.

### β-TRCP controls CYLD protein stability

**Figure 2:**
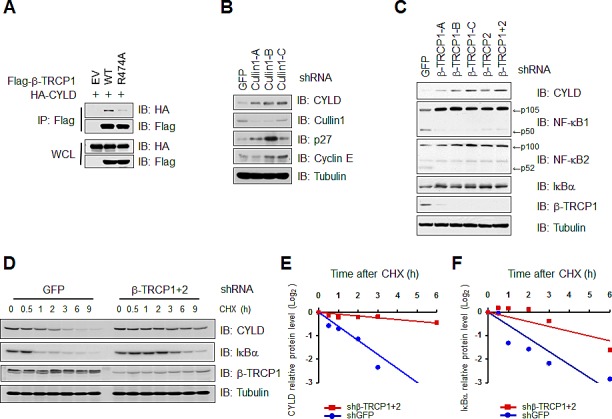
β-TRCP controls CYLD protein stability (A) IB analysis of WCL and IP derived from 293T cells transfected with HA-CYLD and empty vector (EV), wild-type (WT) or R474A mutant β-TRCP1, as indicated. (B) IB analysis of WCL derived from 293T cells infected with shRNA constructs against GFP, Cullin1 (three independent lentiviral Cullin1-targeting shRNA constructs namely, -A, -B, -C), followed by selection with 1 μg/ml puromycin for 72 hours to eliminate non-infected cells. (C) IB analysis of WCL derived from HeLa cells infected with shRNA constructs against GFP, β-TRCP1 (three independent lentiviral β-TRCP1-targeting shRNA constructs β-TRCP1-A, β-TRCP1-B, β-TRCP 1-C), β-TRCP2 or β-TRCP1+2 (shRNA against both β-TRCP1 and 2 isoforms), followed by selection with 1μg/ml puromycin for 72 hours to eliminate non-infected cells. (D) To measure CYLD protein half-life changes, the RAW264.7cells were infected with the indicated shRNA followed by selection with 1 μg/ml puromycin for 72 hours to eliminate non-infected cells. Afterwards, cells were split into 6 cm dishes, treated with 100μg/ml cycloheximide (CHX) and harvested at indicated time points. WCL were prepared and IB analyses were performed with the indicated antibodies. (E) Quantification of the CYLD band intensities in (D). CYLD band intensity was normalized to tubulin, and then normalized to the t = 0 controls. (F) Quantification of the IκBα band intensities in (D). IκBα band intensity was normalized to tubulin, and then normalized to the t = 0 controls.

To demonstrate that CYLD is a *bona-fide* substrate of SCF^β-TRCP^, we next examined whether the interaction of β-TRCP with CYLD is through the substrate recognition domain of β-TRCP. Utilizing the β-TRCP (R474A) mutant that is deficient in binding its substrates for recruitment to the SCF complex [33], we observed a significantly reduced ability of β-TRCP (R474A) to bind CYLD (Figure 2A), supporting a specific interaction between SCF^β-TRCP^ and CYLD. Furthermore, the observation of elevated CYLD protein levels after depleting endogenous Cullin-1 or β-TRCP by multiple shRNAs suggested that CYLD is a potential physiological substrate for SCF^β-TRCP^ (Figures 2B-C and Supplementary Figures 2A-C). Notably, depletion of endogenous β-TRCP1 resulted in a significant increase in the half-life of CYLD and a representative β-TRCP substrate IκBα (Figures 2D-F). Taken together, these results indicated that SCF^β-TRCP^ might negatively regulate the protein stability of CYLD.

### IKK promotes CYLD ubiquitination and subsequent degradation

**Figure 3:**
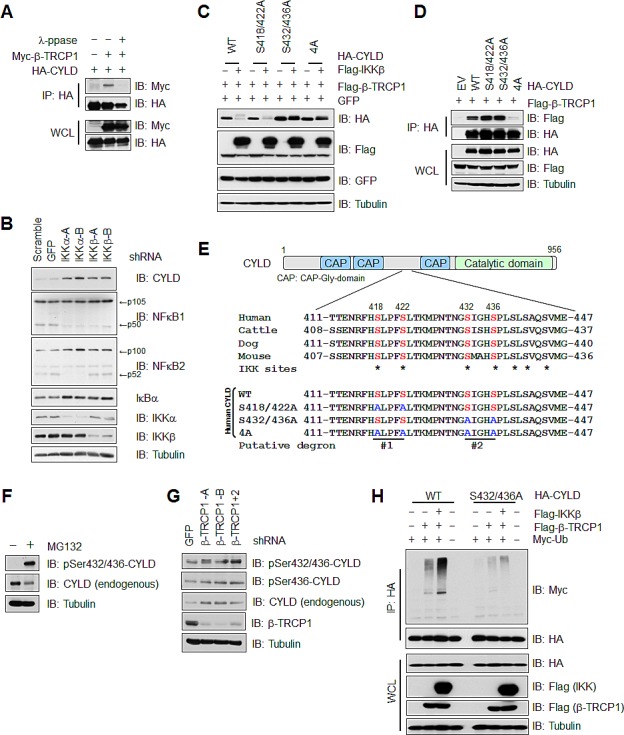
IKK promotes CYLD ubiquitination and subsequent degradation (A) IB analysis of WCL and IP derived from 293T cells transfected with HA-CYLD and Myc-β-TRCP1 constructs. Where indicated, cell lysates were pre-treated with λ-phosphatase (λ-ppase) before the IP procedure. (B) IB analysis of WCL and IP derived from 293T cells transfected with shRNA constructs against IKKα, or IKKβ.(C) IB analysis of WCL derived from 293T cells transfected with Flag-β-TRCP1, Flag-IKKβ and WT, S418/422A, S432/436A, or S418/422/432/436A(4A) mutant CYLD constructs. (D) IB analysis of WCL and IP derived from 293T cells transfected with Flag-β-TRCP1 and wild-type (WT), S418/422A, S432/436A, or S418/422/432/436A (4A) mutant CYLD constructs. (E) Schematic structure of CYLD protein and alignment of the reported phosphorylation sites by IKK that may be recognized by β-TRCP. The CYLD mutants that are used in this study are also indicated. (F) IB analysis of WCL derived from 293T cells treated with 15 μM MG132 or untreated control cells. (G) IB analysis of WCL derived from 293T cells infected with lenti-viral shRNA constructs against GFP, β-TRCP1 (two independent shRNA, -A and -B) or β-TRCP1+2 (shRNA against both β-TRCP1 and 2 isoforms). (H) IB analysis of WCL and IP derived from 293T cells transfected with Flag-β-TRCP1, Myc-Ubiquitin, Flag-IKKβ and HA-tagged WT or S432A/S436A mutant CYLD constructs as indicated.

Proper phosphorylation of substrates proteins by specific kinase(s) is critical for SCF^β-TRCP^ to recognize its substrate(s) for subsequent ubiquitination and degradation [15]. Consistent with a critical role of substrate phosphorylation for recognition by SCF^β-TRCP^, phosphatase treatment led to a marked reduction in interaction between CYLD and SCF^β-TRCP^ (Figure 3A). As IKK-mediated phosphorylation of CYLD has been reported to negatively regulate CYLD enzymatic activity [34], we further explored whether IKK is also involved in regulating the degradation of CYLD by SCF^β-TRCP^. Notably, depletion of endogenous IKKs by multiple shRNAs against IKKα or IKKβ led to an accumulation of CYLD protein in 293T cells (Figure 3B). On the contrary, ectopic expression of IKK significantly reduced CYLD protein abundance in 293T cells ectopically expressing HA-tagged CYLD (Figure 3C). These data therefore indicated a possible role for IKK in promoting the degradation of CYLD. Notably, among the four reported IKK phosphorylation sites [34] (S418, S422, S432 and S436) that may create two putative β-TRCP binding motifs (Figure 3E), mutating both phospho-degrons of CYLD abolished the interaction between CYLD and β-TRCP (Figures 3D-E). However, mutating putative degron #2, but not degron #1, led to a resistance to IKK/SCF^β-TRCP^-mediated degradation of CYLD, arguing that Ser432 and Ser436 serve as the dominant phospho-degron for CYLD degradation by SCF^β-TRCP^ and IKK (Figure 3C). Although this identified phospho-degron motif (GSIGHS) within CYLD is not consistent with the canonical β-TRCP degron (DSGXXS), it does resemble a reported non-canonical degron found within p53 that also contains IKK phosphorylation sites, and other known β-TRCP substrates including Mcl-1, PHLPP1 and Gli2 (Supplementary Figure 3A) [35-38]. To further demonstrate a critical role of the phosphorylation of Ser432 and Ser436 in controlling CYLD protein stability, we developed phosphorylation-specific antibodies that recognize pSer436-CYLD or pSer432/pSer436-CYLD (Supplementary Figures 3B-C). Using these antibodies, we detected an elevation of pSer436-CYLD and pSer432/pSer436-CYLD after blocking proteasome activity by MG132 (Figure 3F), or by depleting endogenous β-TRCP (Figure 3G) in 293T cells. These results support the notion that phosphorylation of CYLD by IKK at Ser432 and Ser436 is associated with β-TRCP-mediated poly-ubiquitination and subsequent degradation of CYLD. In further support of this finding, mutating Ser432 and Ser436 to Alanine abolished IKK-dependent, β-TRCP-mediated poly-ubiquitination of CYLD in 293T cells (Figure 3H). Together, these results demonstrate that IKK-mediated phosphorylation of Ser432 and Ser436 might play a pivotal role in triggering SCF^β-TRCP^-dependent CYLD poly-ubiquitination.

### β-TRCP regulates osteoclast differentiation in part through the β-TRCP/CYLD signaling pathway

**Figure 4:**
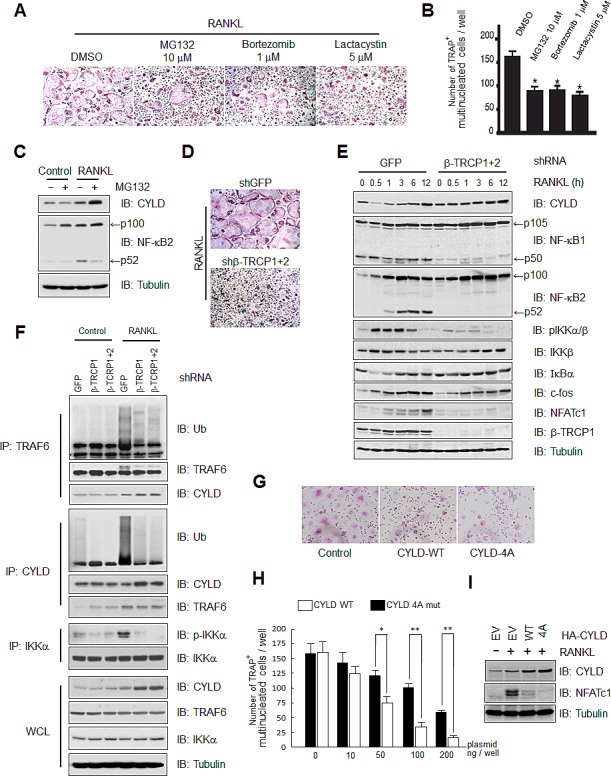
β-TRCP regulates osteoclast differentiation in part through modulating CYLD abundance to influence the activation of NF-κB signaling pathway (A) Tartrate resistant acid phosphatase (TRAP) positive multinucleated cells differentiated from bone marrow macrophage cells derived from bone marrow cells in the presence of 100 ng/ml RANKL for 3 days, followed by treatments with DMSO (control), MG132 (10 μM), Bortezomib (1 μM) or Lactacystin (5 μM). (B) TRAP positive multinucleated cells in each well of (A) were counted under microscopes. Data represents mean ± SD (n=3, *p<0.001 by *Student*'s *t* test). (C) IB analysis of WCL derived from bone marrow macrophage cells treated or non-treated with RANKL (100 ng/ml RANKL for 3 days) in the presence or absence of 10 μM MG132, as indicated. (D) TRAP positive multinucleated cells in the control or β-TRCP1 and β-TRCP2 double knockdown bone marrow precursor cell line in the presence of 100 ng/ml RANKL for 12 hours. (E) IB analysis of WCLs derived from the control or β-TRCP1+/2 knockdown bone marrow precursor line in the presence of 100 ng/ml RANKL for the indicated time periods. (F) *In vivo* ubiquitination assay was performed using RAW264.7 cells infected with shRNA lentiviral vector against GFP, β-TRCP1, and β-TRCP1+2 (shRNA against both β-TRCP1 and 2 isoforms) followed by selection with 1 μg/ml puromycin for 72 hours to eliminate the non-infected cells. Then cells were stimulated with 20 ng/ml RANKL and harvested for IP. (G-I) RAW cells transfected with EV, HA-CYLD-WT, or -4A expression plasmid were treated with 100 ng/ml RANKL for 3 days, and fixed for TRAP staining for osteoclasts (G). TRAP positive multinucleated cells were counted. Data shown are the numbers of TRAP positive multinucleated cells per well of 96 well cell culture plate. Data represents mean ± SD (n=3, *p<0.05, **p<0.001 by *Student*'s *t* test) (H). IB analysis of WCL derived from the indicated RAW cells was presented in (I).

Previous studies have identified that RANKL stimulates osteoclast differentiation through the calcium/NFAT, TRAF6/NF-κB and c-fos signaling pathways [9-12]. Given the critical role of TRAF-mediated K63-ubiquitination as well as the negative feedback role of the CYLD deubiquitinase in modulating osteoclast differentiation, we decided to examine whether proteasome-mediated degradation pathways were important for RANKL-induced osteoclastogenesis. To this end, we treated bone marrow macrophage cells with multiple proteasome inhibitors bortezomib, MG132 and lactacystin, and found that RANKL-induced osteoclastogenesis was blocked by each of these inhibitors (Figures 4A-B). Furthermore, MG132 treatment led to the accumulation of CYLD protein under RANKL-stimulated condition in which CYLD mRNA level is upregulated during the RANKL-induced osteoclast differentiation process with constitutive activation of NF-κB signaling (Figure 4C). Notably, elevated CYLD levels in MG132 treated samples correlate with a reduction of the processed, active form, of p52 and elevation of the non-processed p100 precursor form of NF-κB2, indicating a suppressive effect of NF-κB signaling due to elevated abundance of the CYLD DUB.

On the other hand, in the absence of RANKL stimulation, there is only basal level activity of NF-κB signaling as suggested by the absence of p100 processing to active p52. Given that CYLD is a well-characterized NF-κB transcriptional target, and in keeping with results obtained from DLD1, HCT116 and COS7 cells (Supplementary Figures 1A and F-G), there are low expression levels of endogenous CYLD and contrary to RANKL-stimulated conditions, CYLD could not be upregulated by MG132 that is largely due to the inhibitory effects of MG132 towards NF-κB signaling. These results indicate that osteoclast differentiation is possibly dependent on the proteasome-dependent protein degradation pathway that will affect the abundance of many key NF-κB regulators including CYLD. Therefore, we went on to explore the possible involvement of β-TRCP in RANKL-induced osteoclast differentiation. To this end, we monitored osteoclast differentiation in control and β-TRCP depleted osteoclast precursor cells. As shown in Figure 4D, compared to shGFP-infected control osteoclast precursor cells, osteoclast differentiation induced by RANKL stimulation was significantly blocked in β-TRCP depleted cells (Figure 4D).

In further support of a critical role for CYLD in RANKL-mediated osteoclast differentiation process, we found that RANKL-dependent CYLD degradation observed at 0.5, 1, and 3 hours post RANKL stimulation, was markedly blocked by knockdown of β-TRCP, and likely due to repression of IKK activation resulting from CYLD stabilization (Figure 4E). As CYLD is a critical factor that regulates osteoclast activation in part through TRAF6 and p62/SQSTM1 deubiquitination [18] (Supplementary Figure 4), these results suggest that β-TRCP signaling pathway may be critical in controlling osteoclastogenesis in part by negative regulation of CYLD stability to influence the NF-κB signaling intensity. In support of this notion, *in vivo* poly-ubiquitination assays demonstrate that depletion of β-TRCP impaired TRAF6 self-ubiquitination likely due to enhancement of TRAF6 deubiquitination by CYLD, concomitant with a reduction in β-TRCP-dependent ubiquitination of CYLD and impairment of auto-phosphorylation of TRAF6-downstream kinase IKKα (Figure 4F). To further demonstrate that the observed suppression of osteoclastogenesis with proteasome inhibitors (Figure 4A) or β-TRCP depletion (Figure 4D) is partly through CYLD stabilization and elevated CYLD activity, we ectopically expressed CYLD-WT or -4A in RAW264.7 osteoclast precursor cells, and conducted an osteoclastogenesis assay. As shown in Figure 4G-I, ectopic expression of both WT and 4A CYLD blocked RANKL-induced osteocrastgenesis. Importantly, the number of differentiated osteoclast cells was significantly lower in non-degradable CYLD mutant (CYLD-4A) expressing cells compare to CYLD-WT expressing cells, which was further confirmed by the western blot analysis of the osteocrast marker protein NFATc1. These results confirmed previously reported results demonstrating that CYLD is a critical negative regulator of osteocrastgenesis [25]. Our results further suggest that β-TRCP plays an important role in modulating osteoclastgenesis in part through promoting ubiquitination-dependent degradation of CYLD. Altogether, these results support the model that CYLD degradation by SCF^β-TRCP^ plays a critical role in governing the timely activation of the NF-κB signaling pathway to control the osteoclast differentiation process (Supplementary Figure 4).

## DISCUSSION

Here we demonstrated that IKK and β-TRCP negatively control CYLD protein stability through phosphorylation-dependent poly-ubiquitination. Recent studies have revealed that various extra-cellular stimuli such as RANKL trigger oligomerization and activation of the E3 ubiquitin ligase TRAF6 by undergoing K63-linked self-ubiquitination, which in turn activate the IKK complex for timely degradation of IκB that turns on NF-κB activity (Supplementary Figure 4). On the other hand, activated NF-κB stimulates transcriptional upregulation of CYLD to antagonize TRAF6 by promoting its K63-linked deubiquitination, presenting a negative feedback loop of NF-κB activation [18]. In our current study, we demonstrated that β-TRCP and IKK could possibly sustain the activation loop in canonical NF-kB signaling pathway in part by downregulating the negative regulator CYLD.

Recent studies have identified important roles of the ubiquitin-proteasome system in osteoclastogenesis [39]. In keeping with these reports, we found that the treatment with multiple proteasome inhibitors such as MG132, bortezomib and lactacystin, reduced RANKL-induced osteoclast differentiation (Figures 4A-B). In accordance with these results, knockdown of β-TRCP led to suppression of osteoclast differentiation (Figure 4D), suggesting a positive role of β-TRCP during osteoclastogenesis. Depletion of β-TRCP has been shown to have a multiple suppressive effect on NF-κB activation including upregulation of IκBα and deficiencies in processing of NF-κB1 and NF-κB2. Here we demonstrated that during the RANKL-dependent signaling pathway, CYLD stabilization by depletion of β-TRCP decreased the ubiquitination of TRAF6 and impaired auto-phosphorylation of the TRAF6-downstream kinase IKKα (Figures 4E-F). These results suggest that β-TRCP participates in multiple layers to positively control the NF-κB signaling pathway, in part via governing the stability of the CYLD deubiquitinase.

Studies using knock-out mouse models have demonstrated that mice deficient in both NF-κB1 and NF-κB2, but not deficient in either one alone, developed osteopetrosis due to defects in osteoclast differentiation arguing the involvement of both canonical and non-canonical NF-κB signaling in this process [40]. Furthermore, TRAF6-deficient mice also exhibit defects in the osteoclast differentiation and functions, leading to osteopetrosis phenotypes [41]. Consistently, CYLD knockout mice displayed an osteolytic phenotype associated with hyper-ubiquitination of TRAF6 and persistent activation of its downstream signaling molecules including NF-κB and ERK [25]. The sustained activation of the NF-κB pathway subsequently leads to enhanced sensitization to RANKL stimulation and hyper-differentiation of osteoclast cells in CYLD deficient mice. Interestingly, the point mutation (P392L) of the CYLD interacting protein p62/SQSTM1, an adaptor molecule required for the CYLD-TRAF6-p62/SQSTM1 complex formation, is reported to predispose individuals to the development of Paget's disease [31]. The primary symptoms of this chronic disorder are an enlarged and misshapen bone that resembles the reported phenotypes of CYLD knockout mice. In this study, we report that β-TRCP depletion significantly decreases TRAF6 ubiquitination that directs osteoclastogenesis. Thus, our findings suggest that dysregulation of the β-TRCP-CYLD signaling pathway may contribute to various bone-related pathogenic phenotypes caused by abnormal osteoclastogenesis, and targeting this pathway will be a potential strategy to develop new therapeutic intervention to treat various osteolytic disorders. The outcomes for patients with myeloma have dramatically improved over the past decade, largely due to the availability of better options for treatment including high-dose therapy, thalidomide, bortezomib (Velcade), and lenalidomide [42, 43]. In some reports, bortezomib treatments have been shown to increase bone mineral density in a myeloma/osteosarcoma mouse model [42, 43]. Our findings will further provide new insights into the molecular mechanism of how to maintain optimal bone mineral density through suppressing the excessive bone resorption process in part by inhibiting osteoclast differentiation.

## METHODS

### Cell Culture

RAW264.7 mouse leukemic monocyte macrophage cell line (ATCC, Manassas, VA) was cultured in alpha-Minimal Essential Medium (αMEM) medium supplemented with 10% FBS. 293T, HeLa, DLD1, HCT116 *and* COS-7 cell lines were cultured in DMEM containing 10% FBS and antibiotics (streptomycin and penicillin).

### Plasmids

pDest-CYLD-HA was obtained from Addgene. CYLD and β-TRCP mutants were generated with QuikChange XL Site-Directed Mutagenesis kit. Short hairpin RNAs (shRNA) lentiviral pLKO vectors against β-TRCP1, β-TRCP2, β-TRCP1+2 and GFP were described as previous [44]. Myc-β-TRCP1, Myc-β-TRCP2, Flag-β-TRCP1 and Flag-β-TRCP1-R474A expression plasmids, and shRNA lentiviral vectors against Cullin-1 were obtained from Dr. J. Wade Harper (Harvard Medical School, Boston, MA.). Myc-Cullin-1, Myc-Cullin-2, Myc-Cullin-3, Myc-Cullin-4A, and Myc-Cullin-5 plasmids were obtained from Dr. J. Decaprio (Dana-Farber Cancer Institute, Boston, MA). Retroviral pSUPER short hairpin RNAs (shRNA) vectors against IKKα and IKKβ were obtained from Addgene.

### Antibodies

Anti-CYLD (D1A10), anti-IKKα, anti-IKKα/β, anti-NFATc1, anti-NF-kB1, anti-NF-kB2, anti-β-TRCP1 (D13F10) and anti-Cullin 1 antibodies were purchased from Cell Signaling Technology. Anti-c-Myc (9E10) and polyclonal anti-HA antibodies (Y-11) were purchased from Santa Cruz Biotechnology. Anti-Tubulin, anti-Vinculin, polyclonal and monoclonal anti-Flag antibodies, peroxidase-conjugated anti-mouse secondary antibody, peroxidase-conjugated anti-rabbit secondary antibody, anti-Flag agarose beads, and anti-HA agarose beads were purchased from Sigma-Aldrich. Monoclonal anti-HA antibody was purchased from Covance. Anti-pSer436-CYLD and pSer432/436-CYLD antibodies were developed in collaboration with Cell Signaling Technology.

### Cell Transfections

For transfection, 5×10^5^ 293T or HeLa cells were cultured in 6 cm dish and transfected with Lipofectamine (Invitrogen) in Opti-MEM (Invitrogen) according to the manufacturer's instruction. Forty-eight hours after transfection, cells were lysed in EBC (50 mM Tris, pH 8.0, 120 mM NaCl, and 0.5% NP-40) buffer supplemented with protease inhibitors (Complete Mini; Roche) and phosphatase inhibitors (phosphatase inhibitor cocktail set I and II; EMD). For half-life experiment, transfected cells were split into 60-mm tissue culture dishes 24 hours after transfection, and treated with 100 μg/ml cycloheximide (CHX; Sigma-Aldrich) next day. At indicated time points, cells were lysed for western blotting analysis. The lenti-virus packaging procedures were described previously [45].

### Immunoblots and Immunoprecipitation

Whole cell extracts were collected and the protein concentrations of the lysastes were measured with Bradford assay reagent (Bio-Rad Laboratories) on a DU-800 spectrophotometer (Beckman Coulter). Samples were resolved by SDS-PAGE and western blotted with the indicated antibodies. For immunoprecipitation, 20 hours post transfection, cells were treated with 10 μM MG132 overnight, and then were harvested for immunoprecipitation procedures. 800 μg of protein lysastes were incubated with the appropriate antibody (1-2 μg) overnight at 4 °C following by the addition of carrier beads. Immunocomplexes were washed five times with NETN buffer (20 mM Tris-Cl, pH 8.0, 100 mM NaCl, 1 mM EDTA, and 0.5% NP-40), and then were resolved by SDS-PAGE and western blotted with indicated antibodies.

### Osteoclastogenesis Assays

Bone marrow-derived macrophages (BMMs) were prepared as osteoclast precursors from 3- to 5-week-old male ddY mice. All procedures to prepare the precursor cells were conducted according to Public Health Service Policy on Humane Care and Use of Laboratory Animals and the Fukuoka Dental College Institutional Animal Care and Use Committee (IACUC). Bone marrow cells obtained from the mouse tibia were suspended in 60-mm-diameter dishes for 16 h in the presence of M-CSF (50 ng/ml) in α-MEM containing 10% fetal bovine serum. Then, non-adherent cells were harvested and further cultured for 2 days with M-CSF (50 ng/ml). The adherent cells, most of which expressed macrophage-specific antigens such as Mac-1, Moma-2, and F4/80, were used as BMMs. BMMs were cultured for 3 days with RANKL (50 ng/ml). The macrophage cell line RAW 264.7 cells were cultured for 3 days with RANKL (20 ng/ml). Cultures were fixed with 3.7% formaldehyde, and osteoclasts were detected by staining for tartrate-resistant acid phosphatase (TRAP). TRAP-positive multinucleated cells containing more than three nuclei were observed under a microscope and counted as differentiated osteoclasts.

### Statistical Analysis

Data shown is representative of three independent experiments. All quantitative data were presented as the mean ± SD as indicated of at least three independent experiments by Student's *t* test for between group differences. The *p* < 0.05 was considered as statistically significant.

## SUPPLEMENTARY FIGURES


