# Supplementary material for: SCFβ-TRCP regulates osteoclastogenesis via promoting CYLD ubiquitination
Source: Oncotarget. 2014 May 14;5(12):4211–21. doi: 10.18632/oncotarget.1971 (PMC4147317; doi:10.18632/oncotarget.1971)
Supplement: Supplementary file 1 [file oncotarget-05-4211-s001.pdf]

SCF$^{\beta\text{-TRCP}}$ regulates osteoclastogenesis via promoting CYLD ubiquitination

Supplementary Material

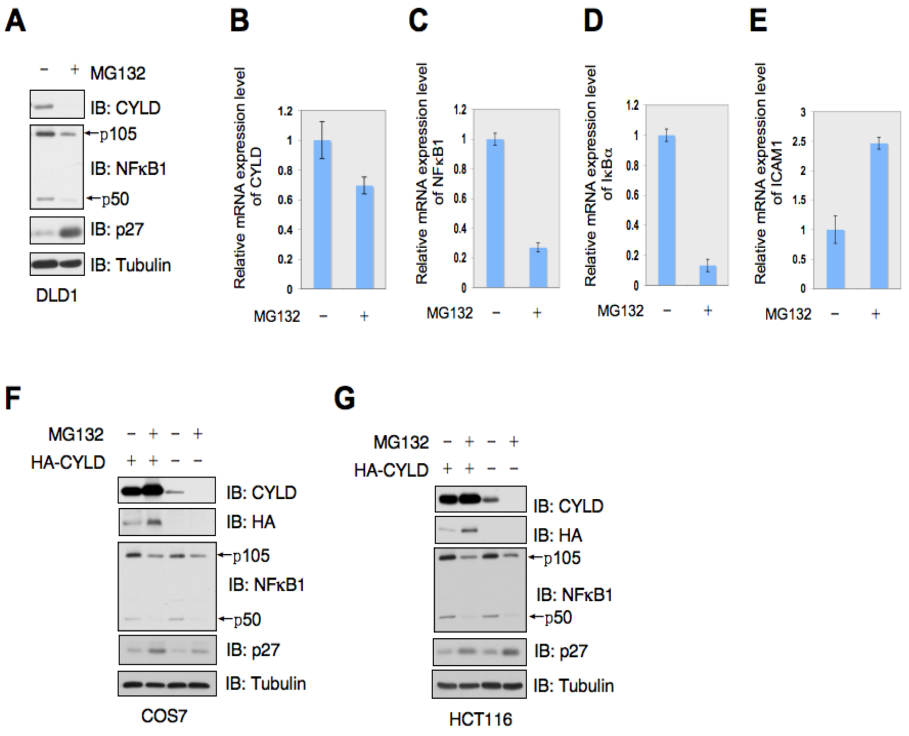

Supplementary Figure 1: Treatment of MG132 leads to the accumulation of exogenous, but not endogenous CYLD protein. (A) Immunoblot (IB) analysis of whole cell lysates (WCL) derived from DLD1 cells with or without MG132 (15 μM) treatment.**(B-E)** Quantification of relative mRNA expression levels of CYLD (**B**), NF-κB1 (**C**), IκBα (**D**) and ICAM1 (**E**) in DLD1 cells with or without MG132 (15 μM) treatment. Data were presented as mean ± SD (n=3).**(F)** IB analysis of WCL derived from COS7 cells transfected with HA-CYLD with or without MG132 treatment.**(G)** IB analysis of WCL derived from HCT116 cells transfected with HA-CYLD with or without MG132 treatment.

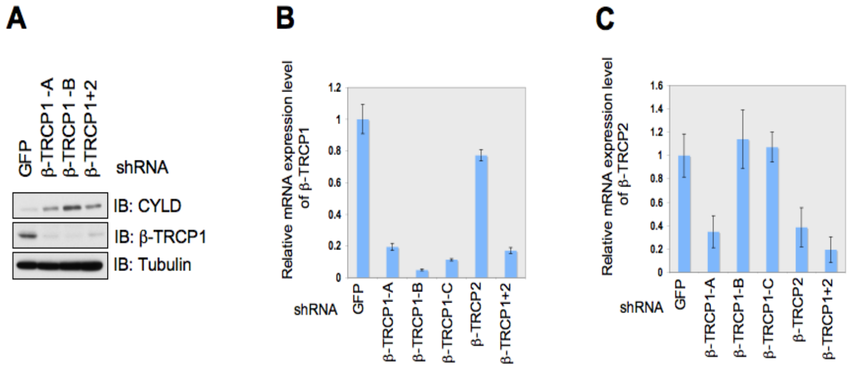

Supplementary Figure 2: Depletion of β-TRCP leads to accumulation of endogenous CYLD protein.(A) IB analysis of WCL derived from 293T cells infected with shRNA constructs against GFP, β-TRCP1 (two independent lentiviral β-TRCP1-targeting shRNA constructs β-TRCP1-A, β-TRCP1-B) or β-TRCP1+2 (shRNA against both β-TRCP1 and 2 isoforms), followed by selection with 1 μg/ml puromycin for 72 hours to eliminate non-infected cells.**(B-C)** Quantification of relative mRNA expression levels of β-TRCP1 (**B**) and β-TRCP2 (**C**) in HeLa cells infected with shRNA constructs against GFP, β-TRCP1 (two independent lentiviral β-TRCP1-targeting shRNA constructs β-TRCP1-A, β-TRCP1-B or β-TRCP1-C), β-TRCP2, or β-TRCP1+2 (shRNA against both β-TRCP1 and 2 isoforms), followed by selection with 1 μg/ml puromycin for 72 hours to eliminate non-infected cells. Data were presented as mean ± SD (n=3).

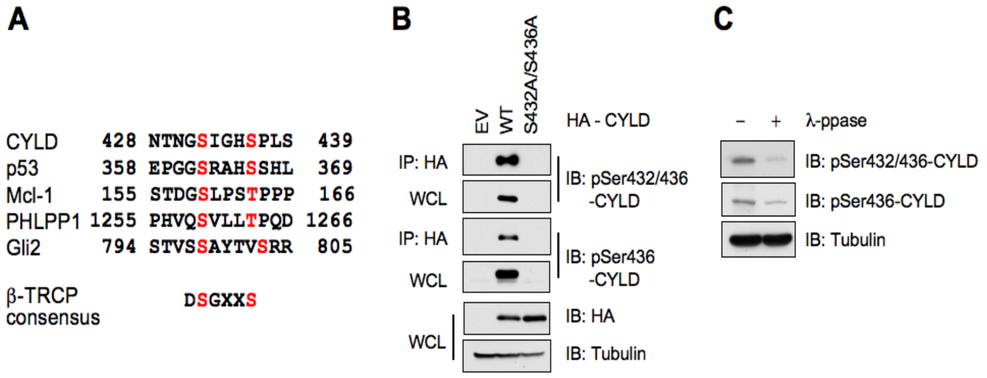

Supplementary Figure 3: β-TRCP recognition motif in CYLD is phosphorylated in 293T cells. (A) Alignment of the identified CYLD and other reported non-canonical β-TRCP phospho-degron sequences. Phosphorylated serine or threonine residues within the phospho-degrons are colored with red. **(B)** IB analysis of WCL and HA-IP derived from 293T cells transfected with wild-type (WT) or S432A/S436A mutant form of CYLD. **(C)** IB analysis of WCL derived from 293T cells. Where indicated, cell lysates were pre-treated with λ-phosphatase (λ-ppase) before denaturing with SDS sample buffer.

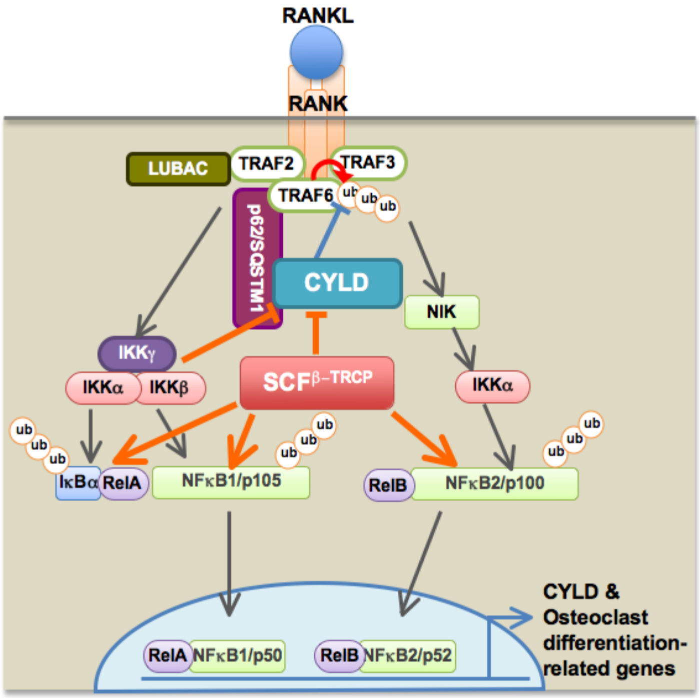

Supplementary Figure 4: A schematic illustration of how SCF$^{\beta\text{-TRCP}}$ participates in regulation of osteoclast differentiation by modulating the activity of the RANKL/NF-κB signaling pathway.

SCF$^{\beta\text{-TRCP}}$ is reported to positively regulate the NF-κB signaling pathway through degrading IκB and processing NF-κB1/2 in osteoclast cells. CYLD degradation by SCF$^{\beta\text{-TRCP}}$ could be another node of positive feedback loop, leading to sustained activation of the NF-κB signaling to influence various cellular processes including osteoclast differentiation.
